# Autophagy in HCV Replication and Protein Trafficking

**DOI:** 10.3390/ijms22031089

**Published:** 2021-01-22

**Authors:** Ja Yeon Kim Chu, Jing-hsiung James Ou

**Affiliations:** Department of Molecular Microbiology and Immunology, Keck School of Medicine, University of Southern California, Los Angeles, CA 90089, USA; jayeonki@usc.edu

**Keywords:** hepatitis C virus, autophagy, mitophagy, autophagosome biogenesis, HCV RNA replication, innate immunity, protein trafficking

## Abstract

Autophagy is a catabolic process that is important for maintaining cellular homeostasis. It is also known to possess other functions including protein trafficking and anti-microbial activities. Hepatitis C virus (HCV) is known to co-opt cellular autophagy pathway to promote its own replication. HCV regulates autophagy through multiple mechanisms to control intracellular protein and membrane trafficking to enhance its replication and suppress host innate immune response. In this review, we discuss the current knowledge on the interplay between HCV and autophagy and the crosstalk between HCV-induced autophagy and host innate immune responses.

## 1. Introduction

Hepatitis C virus (HCV) is an important human pathogen that can cause severe liver diseases including acute and chronic hepatitis, cirrhosis, and hepatocellular carcinoma (HCC). According to the report of World Health Organization (WHO), there are approximately 71 million people in the world who are chronically infected by this virus, resulting in nearly 400,000 deaths annually, mostly due to liver cirrhosis and HCC. There are 1.75 million new HCV infections every year as per the estimation of 2015. HCV is transmitted primarily via intravenous drug use, blood transfusions, and poorly sterilized medical equipment. There is no vaccine available to prevent HCV infection. However, highly efficacious direct acting antivirals (DAAs) against HCV have been developed and used to treat HCV patients with a curing rate of more than 95%. Unfortunately, most HCV patients are not aware of this viral infection until severe symptoms have developed, and patients with advanced fibrosis or cirrhosis still have a high risk of developing HCC even after a successful treatment with DAAs [1]. The high cost of DAAs has also limited their use, and the appearance of drug-resistant mutants is posing new challenges [2].

## 2. HCV Lifecycle

HCV is a hepatotropic virus that belongs to the *Hepacivirus* genus of the *Flaviviridae* family [3]. It is an enveloped virus with a single-stranded RNA genome. The HCV genome is about 9.6-kb in length and has a positive polarity. It encodes a polyprotein with a length of slightly over 3000 amino acids. This polyprotein is translated using an internal ribosomal entry site (IRES) located near the 5′-end of the viral genome [4]. The HCV polyprotein is co- and post-translationally processed by cellular (i.e., signal peptidase and signal peptide peptidase) and viral proteases (i.e., NS2 and NS3 proteases) to generate 10 viral proteins [5]. The viral structural proteins, which are the core protein (i.e., the capsid protein) and E1 and E2 envelope proteins, are required for the formation of mature HCV particles. Most of the nonstructural proteins, which include NS3, NS4A, NS4B, NS5A, and NS5B, are required for the replication of the viral genomic RNA [6]. In addition, p7 and several of the nonstructural proteins such as NS2, NS3, NS4A, and NS5A are also involved in the assembly of the viral particles. Many of the HCV proteins also have regulatory functions. The infection of hepatocytes by HCV is initiated by the interactions between HCV and the host co-receptors, including CD-81, scavenger receptor-BI (SR-BI), claudin-1, and occludin [7]. These interactions are followed by the internalization of HCV into hepatocytes by endocytosis and the subsequent release of the HCV genomic RNA into the cytosol. The released HCV genomic RNA will serve as the mRNA for the synthesis of HCV proteins. The HCV nonstructural proteins NS3, NS4A, NS4B, NS5A, and NS5B will form a replication complex to mediate HCV RNA replication, which takes place on membranous structures [8,9]. The assembly of HCV core particles is associated with lipid droplets in close proximity to endoplasmic reticulum (ER) membranes [10]. After the packaging of the HCV genomic RNA, core particles interact with viral envelope proteins to form mature viral particles, which are subsequently released from infected cells.

## 3. Autophagy

Many reports have indicated that HCV can induce autophagy (i.e., macroautophagy) to enhance its own replication [11,12]. Autophagy is a catabolic process that is important for maintaining cellular homeostasis. It removes protein aggregates and damaged organelles from cells. Autophagy can be initiated by many stimuli including nutrient starvation, oxidative stress, ER stress, and microbial infections [13]. This process begins with the formation of membrane crescents, called phagophores or isolation membranes, in the cytoplasm. The membrane of phagophores will subsequently extend to form enclosed double-membrane vesicles, known as autophagosomes. Autophagosomes mature by fusing with lysosomes to form autolysosomes, in which the cargos of autophagosomes are degraded by lysosomal enzymes for recycling [14]. Alternatively, autophagosomes may also fuse with multivesicular bodies (i.e., late endosomes) to form amphisomes to positively or negatively regulate the release of exosomes from cells [15]. More than 30 autophagy-related proteins (ATGs) and two ubiquitin-like conjugation systems that are important for autophagy have been identified. Autophagy is initiated after the activation of the class III phosphatidylinositol-3-kinase (PI3KC3), which catalyzes the formation of phosphatidylinositol-3-phosphate (PI3P) to trigger the formation of the pre-autophagosomal structure (also known as the phagophore assembly site) (PAS) [16]. One ubiquitin-like conjugation system is involved in the covalent linking of ATG5 and ATG12, which will recruit ATG16 to form a complex. This ATG5–ATG12–ATG16 complex is important for the formation of phagophores. A second ubiquitin-like conjugation system is involved in the coupling of the microtubule-associated protein light-chain 3 (LC3) to phosphatidylethanolamine (PE), a phospholipid. This lipidation allows LC3 to localize to autophagosomal membranes and is important for the formation of autophagosomes. LC3 is de-lipidated by ATG4 after the maturation of autophagosomes and released back into the cytosol. It can also be degraded by lysosomal enzymes if it is localized to the inner membrane of autophagosomes [14]. Autophagy can function as a cell defense mechanism by eliminating intracellular microbial pathogens in a process known as xenophagy [17,18]. However, some microbial pathogens including viruses have developed mechanisms to subvert autophagy and use it to support their own replications. HCV has been shown to induce autophagy to enhance its own replication [11,19,20,21].

## 4. Mechanism of HCV-Induced Autophagy

HCV infection induces autophagy in its host cells, including hepatoma cells, primary human hepatocytes, and hepatocytes of infected individuals [19,20,22]. This is evidenced by the increase of lipidation of LC3 and the accumulation of autophagic vacuoles in HCV-infected cells. HCV induces autophagy via multiple pathways [11]. It can induce autophagy indirectly and directly. HCV has been shown to induce the ER stress and activate the unfolded protein response (UPR) [20,23,24,25,26]. The accumulation of unfolded or misfolded proteins in the ER will induce the ER stress, leading to the activation of the activating transcription factor 6 (ATF6), the inositol-requiring enzyme 1 (IRE1), and the double-stranded RNA-activated protein kinase-like ER kinase (PERK) to trigger downstream signaling pathways, which are collectively known as the UPR. The UPR is important for HCV-induced autophagy, as the silencing of ATF6, IRE1, or PERK significantly inhibits HCV-induced autophagy [20,23,27]. Further analysis indicated that the ER stress induced by HCV could inhibit AKT, also known as protein kinase B (PKB), and a negative regulator of tuberous sclerosis complex (TSC), to result in the inhibition of the mammalian target of rapamycin complex I (mTORC1). This led to the activation of the UNC-51-like kinase 1 (ULK1) and the induction of autophagy [24]. A separate study of the same group also indicated that the HCV core protein by itself was sufficient to induce the ER stress, although it activated only PERK and ATF6 without activating IRE1 [27]. The activation of PERK led to the induction of the ATF4 transcription factor and the DNA damage-inducible transcript 3 protein (DDIT3, also known as CHOP). ATF4 upregulates the expression of ATG12 whereas CHOP binds to nucleotides −293 to −99 of the LC3B promoter to stimulate the expression of LC3B to induce autophagy [27]. It should be noted that in a separate study conducted by Mohl et al. [28], it was found that the lipidation of LC3 preceded detectable UPR responses and that the IRE1 knockdown did not affect the lipidation of LC3 in HCV-infected cells. Thus, Mohl et al. questioned the role of the ER stress in HCV-induced autophagy. The reason for this discrepancy of results is not clear. However, in the study by Mohl et al., the LC3 lipidation was detected at as early as 4 h post-HCV infection [28], which was in sharp contrast to the studies conducted by Huang et al., who used the same HCV JFH1 strain for the infection studies and did not detect a significant increase of LC3 lipidation until 4 days after infection [24]. In the studies of Huang et al., the authors used UV-inactivated HCV as the negative control in their infection studies to rule out the possible nonspecific effect of the inoculum. However, such control was not included in the studies of Mohl et al. and hence the possible induction of autophagy by the nonspecific effect of the inoculum could not be ruled out. It is also possible that this early increase of lipidated LC3 detected by Mohl et al. was due to the induction of Rubicon, which suppresses the maturation of autophagosomes and hence increases the level of lipidated LC3 (see below).

HCV has also been known to induce autophagy via the induction of oxidative stress [29]. HCV can induce oxidative stress through multiple mechanisms including the induction of chronic liver inflammation, iron overload, and liver injury [30]. The activities of many of its gene products that include core, E1, E2, NS4B, and NS5A proteins can also induce oxidative stress [31]. High levels of reactive oxygen species (ROS) induced by HCV cause the phosphorylation of serine-349 of the p62 sequestosome protein to induce autophagy [29]. p62 interacts with LC3 and is important for the delivery of proteins to autophagosomes for their eventual degradation in autolysosomes. The phosphorylation of p62 at serine-349 increases the affinity of p62 to Keap1, thereby disrupting the interaction between Keap1 and the nuclear factor E2-related factor 2 (Nrf2). This leads to the nuclear localization of Nrf2 and the activation of its target genes including many antioxidant genes. However, whether HCV can activate Nrf2 is controversial. While one report indicated that it could [32], others have indicated that Nrf2 was retained in the cytoplasm in association with the HCV RNA replication complex and failed to localize to the nucleus to activate its target genes [29,33]. How ROS induced by HCV mediates the serine-349 phosphorylation of p62 is unclear. A previous study indicated that hVps34, the catalytic subunit of PI3KC3, could enhance the interaction between protein kinase C-δ (PKC-δ) and p62 for the phosphorylation of p62 at serine-349 in breast cancer cells [34]. However, whether this is also the case in HCV-infected cells remains to be determined, as previous studies indicated that hVps34 was dispensable for HCV-induced autophagy [35,36].

In addition to indirectly inducing autophagy, HCV can also induce autophagy directly via the activities of its proteins. The expression of the HCV NS3-NS5B nonstructural polyprotein was sufficient to induce double-membrane vesicles that resembled autophagosomes [37]. Further expression studies of individual HCV nonstructural proteins indicated that HCV NS4B protein was sufficient to induce the UPR, the lipidation of LC3, and autophagic vacuoles [38]. The NS3/4A complex, and NS5A and NS5B individually were also able to induce autophagic vacuoles, although the effects of the latter two were much less prominent [27,38]. HCV NS3 interacts with immunity-related GTPase family M protein (IRGM) [39], which is a member of the small GTPase family and can interact with multiple autophagy-associated proteins such as ATG5 and ATG10. IRGM is critical for HCV-induced autophagy, as its depletion suppressed HCV-induced autophagy. HCV NS3 may interact with ATG5 and ATG10 via IRGM to induce autophagy. Alternatively, as IRGM mediates the dephosphorylation of serine-757 of ULK1, which is a kinase important for the initiation of autophagy, in HCV-infected cells [40], it is also possible that this effect of IRGM on ULK1 is triggered by the binding of HCV NS3 to IRGM, leading to the initiation of autophagy. Further studies will be required to test these possibilities. HCV NS4B can induce the expression of Rubicon, which suppresses the fusion between autophagosomes and lysosomes (see below), and may increase the levels of lipidated LC3 and autophagosomes through this mechanism [38]. NS4B has also been shown to interact with Beclin-1, hVps34, and Rab5 to modulate autophagy [11]. Beclin-1 is a component of the PI3KC3 complex. However, as the silencing of hVps34, the catalytic subunit of PI3KC3, and the inhibition of PI3KC3 with 3-methyladenine did not abolish autophagy induced by HCV [35], whether the interaction between NS4B, Beclin-1, and hVps34 plays any role in HCV-induced autophagy remains to be determined. In this regard, it is interesting to note that the HCV p7 ion channel protein had also been found to bind to Beclin-1 without inducing any autophagic response [41].

**Figure 1:**
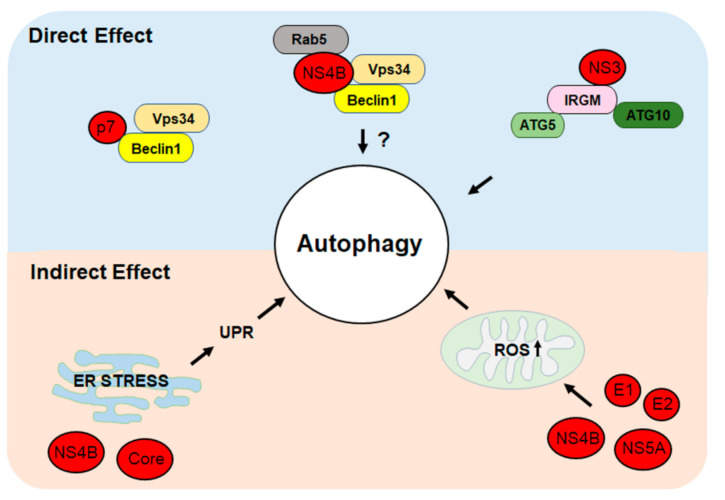
Direct and indirect mechanisms of hepatitis C virus (HCV)-induced autophagy. HCV proteins can directly induce autophagy by interacting with cellular proteins that regulate autophagy. HCV can also indirectly induce autophagy by inducing the ER stress to trigger the unfolded protein response (UPR) or by inducing the production of reactive oxygen species (ROS) and oxidative stress. See text for details.

The molecular mechanisms of HCV-induced autophagy, including direct and indirect effects, are illustrated in Figure 1.

## 5. Biogenesis of Autophagosomes Induced by HCV

**Figure 2:**
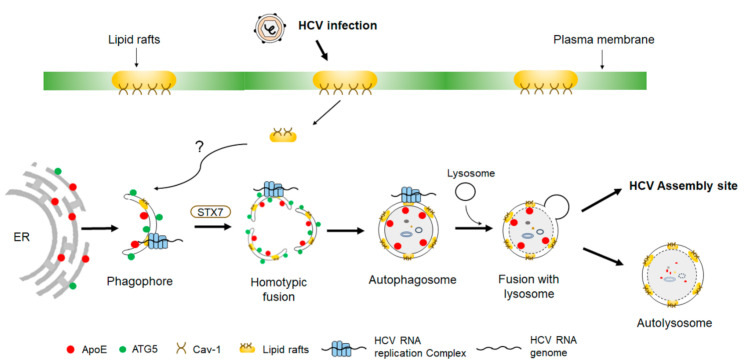
Biogenesis of autophagosomes induced by HCV. Phagophores induced by HCV originate from the ER membranes. The HCV RNA replication complex as well as its associated lipid rafts and caveolin-1 (Cav-1) become associated with phagophores through an unknown mechanism. Phagophores subsequently undergo homotypic fusion in a process dependent on syntaxin 7 (STX7) to form autophagosomes. During these processes, apolipoprotein E (ApoE) becomes associated with autophagosomes and is delivered by autophagosomes to the HCV assembly site to interact with the HCV E2 envelope protein. Some of the autophagosomes may also fuse with lysosomes to form autolysosomes to result in the autophagic degradation of ApoE.

The biogenesis of autophagosomes is often initiated at an ER subdomain enriched in phosphatidylinositol-3-phosphate (PI3P), a product of PI3KC3. This subdomain is called the omegasome, which serves as the PAS [42]. As mentioned above, HCV infection induces the accumulation of autophagosomes in its host cells. The induction of autophagosomes was found in cells transfected with the HCV genomic RNA or infected with HCV, and in cells harboring the replicating HCV subgenomic RNA replicon [23,35,38,43,44,45]. It had previously been reported that the double-FYVE domain-containing protein 1 (DFCP1), which binds to PI3P to initiate the biogenesis of autophagosomes, is important for HCV RNA replication [36]. As the silencing of DFCP1 also led to the reduction of LC3 lipidation, this report supports the involvement of omegasomes in the initiation of autophagosomes induced by HCV. However, as mentioned above, PI3KC3 is dispensable for HCV-induced autophagy [35,36]. Thus, DFCP1 may participate in the biogenesis of HCV-induced autophagosomes via a noncanonical pathway that does not involve PI3P. By conducting live-cell imaging, our recent studies indicated that phagophores induced by HCV originated from the ER [46]. They then underwent homotypic fusion to generate autophagosomes via a pathway dependent on the SNARE protein syntaxin 7 [46] (Figure 2). Curiously, the time required for phagophores to progress to autophagosomes in HCV-infected cells was approximately 30 min, whereas that for autophagy induced by nutrient deprivation took less than 10 min [46]. The reason why the biogenesis of autophagosomes in HCV-infected cells requires a much longer period of time is unclear, possibly being due to the involvement of homotypic fusion of phagophores and/or other biological processes specific to HCV. HCV infection causes the fragmentation of Golgi membranes in an IRGM-dependent manner [40]. Interestingly, these fragmented Golgi membranes were found to colocalize with LC3 puncta (i.e., autophagosomes), the ER marker, and the replicating HCV RNA, suggesting that Golgi membranes may also be involved in the biogenesis of autophagosomes [40].

Multiple studies have indicated that the fusion of autophagosomes with lysosomes is delayed in HCV-infected cells, resulting in the accumulation of autophagosomes in the early stage of HCV infection [20,35,38,47,48]. This delayed maturation of autophagosomes in HCV-infected cells was found to be due to the differential induction of Rubicon and UVRAG proteins by HCV [38,48]. Rubicon negatively regulates the maturation of autophagosomes, whereas UVRAG positively regulates it. In the early stage of HCV infection, Rubicon was upregulated, which inhibited the fusion between autophagosomes and lysosomes. However, in the late stage of infection, UVRAG was also upregulated to overcome the inhibitory effect of Rubicon, resulting in the maturation of autophagosomes and the completion of the autophagic flux [38]. Jones-Jamtgaard et al. also reported that Arl8b, an Arf-like GTPase that localizes to lysosomes and regulates the autophagic flux, was redistributed by HCV to peripheral locations to suppress the autophagosome–lysosome fusion [47]. These studies indicated that HCV could use different mechanisms to delay the maturation of autophagosomes and suppress the autophagic flux. This temporal regulation of the autophagic flux allows the accumulation of autophagosomes in the early stage of HCV infection and is beneficial to HCV replication (see below).

## 6. Role of Autophagy in HCV Replication and Maturation

Autophagy has been shown to play a positive role in HCV replication in many studies [19,20,21,23,28,35,39,44,45]. Dreux et al. reported that autophagy was required for the translation of incoming HCV RNA immediately after infection, but it was not essential for HCV replication once the infection had been established [21]. This proposed effect of autophagy on HCV RNA translation was not consistent with earlier reports [19,20,22] and had been challenged by others, which indicated that autophagy was required for the efficient HCV RNA replication and the production of progeny HCV particles [23,35,49]. Autophagy had been shown to play an important role in HCV RNA replication. Sir et al. demonstrated that nascent HCV RNA labeled with bromouridine triphosphate colocalized with autophagosomes, indicating the association of the HCV RNA replication complex with autophagosomes [20,35]. This finding was further supported by our observation that lipid rafts, which are subdomains of plasma membranes enriched in cholesterol and sphingolipids and essential for HCV RNA replication [50], were localized to autophagosomes in HCV-infected cells [51] (Figure 2). In contrast, lipid rafts were not associated with autophagosomes induced by nutrient starvation or rapamycin and remained mostly associated with plasma membranes [51]. How HCV rerouted lipid rafts from plasma membranes to autophagosomes is unclear and may be related to the unique mechanisms that HCV uses to induce autophagy. For example, nutrient starvation or rapamycin inhibits mTORC1 to induce autophagy, whereas HCV induces autophagy via the induction of the ER stress and oxidative stress and the biological activities of its proteins, which may disrupt the trafficking of the scaffold proteins of lipid rafts including caveolin-1 and annexin A2 to plasma membranes to result in their localization to autophagosomes. The most direct piece of evidence, which demonstrated that autophagosomes could serve as the platform for HCV RNA replication, came from our recent studies, in which we found that HCV-induced autophagosomes purified from cells could mediate HCV RNA replication in vitro, and the depletion of cholesterol, a major component of lipid rafts, from autophagosomes would abolish HCV RNA replication [51]. The assembly of the HCV RNA replication complex on autophagosomal membranes was initiated prior to the formation of autophagosomes and likely during the formation of phagophores or earlier, as the suppression of syntaxin 7 expression with siRNA to block the homotypic fusion of phagophores and the generation of autophagosomes had no effect on HCV RNA replication in HCV-infected cells, and phagophores purified from HCV-infected cells could also mediate HCV RNA replication in vitro [46] (Figure 2). Mohl et al. found a transient association of the HCV NS5A protein with DFCP1 in HCV-infected cells [36]. Their finding would be consistent with the assembly of the HCV RNA replication complex during the formation of omegasomes [36].

In addition to supporting HCV RNA replication, autophagy has also been shown to play a role in HCV maturation and release. Apolipoprotein E (ApoE) interacts with the HCV E2 envelope protein and plays an important role in the maturation of HCV and the increase of its infectivity [52,53]. It is partially localized to autophagosomes and degraded in autolysosomes. The suppression of autophagic protein degradations increased the level of ApoE in autophagosomes and enhanced its colocalization with the HCV E2 envelope protein and the production of infectious HCV particles [54]. Autophagosomes play an important role in the trafficking of ApoE to promote its interaction with the HCV E2 protein, as the suppression of the expression of ATG7, a protein essential for the formation of autophagosomes, reduced the colocalization of ApoE with E2 and the production of infectious progeny HCV particles [54]. Other reports also indicated that the suppression of key regulators of autophagy such as ATG7 and Beclin-1 or the inhibition of autophagy with chemicals reduced the release of HCV from cells [22,29,55].

HCV can stimulate lipogenesis and the accumulation of lipid droplets, which are important for the morphogenesis of HCV particles [56]. Interestingly, lipid droplets were also frequently found to colocalize with HCV-induced autophagosomes [57]. As the inhibition of autophagy led to the increase of cholesterol deposits in HCV-infected cells [56], these observations indicated that HCV-induced autophagy could also remove lipid droplets. The biological significance of this removal of lipid droplets by HCV-induced autophagy, a process known as lipophagy, in the morphogenesis of HCV particles remains to be determined.

## 7. HCV and Mitophagy

Mitophagy is the selective removal of mitochondria by autophagy [58]. In mammalian cells, mitophagy is regulated by two key protein factors PINK1 and PARKIN. PINK1 is a serine/threonine kinase. It is normally transported into mitochondria and cleaved at the N-terminus by the mitochondrial protease PARL at the mitochondrial inner membrane. The N-terminally truncated PINK1 is subsequently released back to the cytosol and degraded by proteasomes [59]. However, when mitochondria are depolarized, the import of PINK1 to the mitochondrial inner membrane is blocked, resulting in its accumulation on the mitochondrial outer membrane where it will recruit PARKIN, an E3 ubiquitin ligase, to ubiquitinate mitochondrial outer membrane proteins to trigger mitophagy. HCV has been shown to induce the autophagic degradation of mitochondria [60,61]. It was found that HCV infection induced the expression of PINK1 and PARKIN and increased the number of autophagosomes and autolysosomes that contained mitochondria [60]. As the silencing of PINK1 or PARKIN hindered HCV replication, mitophagy plays a positive role in HCV replication [60]. Further studies by the same group indicated that HCV could also induce the expression of dynamin-related protein 1 (Drp1) and its phosphorylation at serine-616 [61]. Drp1 is a GTPase and plays an important role in mediating mitochondrial fission, which is often coupled with mitophagy. HCV could also induce the expression of Drp1 receptor mitochondrial fission factor (MFF) [61]. These effects of HCV on Drp1 and MFF led to the localization to Drp1 to mitochondria to trigger mitochondrial fission and mitophagy. Silencing of Drp1 did not affect HCV replication, but it suppressed the release of progeny HCV particles from infected cells with a concomitant reduction of cellular glycolysis and ATP levels. It also increased cytochrome C release from mitochondria and enhanced apoptotic signaling. Thus, HCV can perturb mitochondrial dynamics by stimulating mitochondrial fission and mitophagy and attenuate apoptosis to promote its persistence and replication. A more recent study indicated that the HCV NS5A protein alone was sufficient to reduce the mitochondrial membrane potential and induce mitochondrial fragmentation and mitophagy [62]. Interestingly, in this particular study, the induction of mitophagy by NS5A appeared to be mediated by ROS, as the treatment of cells expressing NS5A with the antioxidant N-acetylcysteine suppressed NS5A-induced mitophagy. We recently discovered that mitophagy could deplete the tumor suppressor p53 in hepatoma cells to increase the level of cells displaying the cancer stem cell properties [63]. Thus, it is conceivable that the persistent replication of HCV in hepatocytes of patients and the perturbation of mitochondrial dynamics and cellular metabolism as well as the induction of stem cell-like properties contribute to HCV pathogenesis, including the development of HCC.

## 8. Suppression of Host Innate Response by HCV-Induced Autophagy

HCV-induced autophagy has been shown to suppress innate immune signaling [64]. The UC-rich sequence located in the 3′ untranslated region of the HCV genomic RNA is recognized by RIG-I, a cytosolic pattern recognition receptor (PRR) that can induce the type-I interferon (IFN) response [64]. It was previously reported that the suppression of autophagy either by silencing the expression of ATG5 or by inhibiting the autophagic protein degradation with chloroquine could induce RIG-I mediated interferon signaling [65]. Ke and Chen found that the silencing of ATG5 expression or the treatment with chloroquine to suppress autophagy in HCV-infected cells could similarly stimulate the expression of type-I IFNs via RIG-I and activate the IFN signaling pathway [23]. In agreement with this finding, Shrivastava et al. [66] reported that the knockdown of Beclin-1 or ATG7 in immortalized human hepatocytes (IHH) infected by HCV led to an increased expression of type I IFNs as well as 2′-5′-oligo-A synthetase-1 (OAS1) and IFI27, which are IFN-stimulated genes (ISGs). In addition, HCV-induced autophagy could also lead to the sequestration of TRAF6 in autophagosomes and its subsequent degradation in autolysosomes [67]. TRAF6 is a member of the tumor necrosis factor receptor-associated factor (TRAF) family and an adaptor molecule of the toll-like receptor (TLR) signaling cascade. Its depletion suppressed the NF-κB signaling pathway and inhibited the production of pro-inflammatory cytokines in HCV-infected hepatocytes [67]. Finally, it was also shown that the silencing of Drp1 to suppress mitochondrial fission and mitophagy also enhanced IFN signaling, leading to the activation of the IFN-stimulated response element in a reporter assay in HCV-infected cells [61]. These results indicated that HCV-induced autophagy and mitophagy could impair innate immune response, which likely plays an important role in HCV persistence in patients.

## 9. Conclusions

Autophagy plays an important role in maintaining cellular homeostasis and can also remove intracellular pathogens. However, HCV has evolved sophisticated mechanisms to exploit this cellular pathway to enhance its own replication. HCV induces autophagy via multiple mechanisms that include the induction of ER stress and oxidative stress and the biological activities of its proteins. It also temporally regulates the autophagic flux, which leads to the accumulation of autophagosomes in the early stage of its infection and induces the localization of lipid rafts to autophagosomes to support its RNA replication. It also uses autophagy to promote the interaction between ApoE and its E2 envelope protein for the production of infectious progeny viral particles and to negatively regulate the host innate immune response. HCV infection also affects mitochondrial dynamics and induces mitophagy to alter cellular metabolism and suppress apoptotic response and IFN signaling. The use of autophagy and mitophagy to suppress host innate immune response is likely an important reason as to why HCV is able to establish chronic infection in the great majority of patients that it infects, and the prolonged perturbation of this important cellular pathway likely also plays an important role in HCV pathogenesis.

## Data Availability

Not applicable.
